# Identification of robust diagnostic and prognostic gene signatures in different grades of gliomas: a retrospective study

**DOI:** 10.7717/peerj.11350

**Published:** 2021-05-11

**Authors:** Jieting Liu, Hongrui Zhang, Jingyun Zhang, Zhitong Bing, Yingbin Wang, Qiao Li, Kehu Yang

**Affiliations:** 1 The First Clinical Medical College, Lanzhou University, Lanzhou, China; 2 Department of Anesthesiology, Lanzhou University Second Hospital, Lanzhou, China; 3 Evidence-based Medicine Center, Lanzhou University, Lanzhou, China; 4 College of Pharmacy, Lanzhou University, Lanzhou, China; 5 School of Public Health and the Second Affiliated Hospital, Zhejiang University School of Medicine, Hangzhou, China; 6 Department of Computational Physics, Institute of Modern Physics, Chinese Academy of Sciences, Lanzhou, China; 7 School of Nuclear Science and Technology, University of Chinese Academy of Sciences, Lanzhou, China; 8 Department of Neurosurgery, Lanzhou University Second Hospital, Lanzhou, China

**Keywords:** Gliomas, Bioinformatics, Gene Signatures, Prognosis, LASSO

## Abstract

**Background:**

Gliomas are the most common primary tumors of the central nervous system. The complexity and heterogeneity of the tumor makes it difficult to obtain good biomarkers for drug development. In this study, through The Cancer Genome Atlas (TCGA) and Chinese Glioma Genome Atlas (CGGA), we analyze the common diagnostic and prognostic moleculer markers in Caucasian and Asian populations, which can be used as drug targets in the future.

**Methods:**

The RNA-seq data from Genotype-Tissue Expression (GTEx) and The Cancer Genome Atlas (TCGA) were analyzed to identify signatures. Based on the signatures, the prognosis index (PI) of every patient was constructed to predict the prognostic risk. Also, gene ontology (GO) functional enrichment analysis and KEGG analysis were conducted to investigate the biological functions of these mRNAs. Glioma patients’ data in the CGGA database were introduced to validate the effectiveness of the signatures among Chinese populations. Excluding the previously reported prognostic markers of gliomas from this study, the expression of HSPA5 and MTPN were examined by qRT-PCR and immunohistochemical assay.

**Results:**

In total, 20 mRNAs were finally selected to build PI for patients from TCGA, including 16 high-risk genes and four low-risk genes. For Chinese patients, the log-rank test p values of PI were both less than 0.0001 in two independent datasets. And the AUCs were 0.831 and 0.907 for 3 years of two datasets, respectively. Moreover, among these 20 mRNAs, 10 and 15 mRNAs also had a significant predictive effect via univariate COX analysis in CGGA_693 and CGGA_325, respectively. qRT-PCR and Immunohistochemistry assay indicated that HSPA5 and MTPN over-expressed in Glioma samples compared to normal samples.

**Conclusion:**

The 20-gene signature can forecast the risk of Glioma in TCGA effectively, moreover it can also predict the risks of Chinese patients through validation in the CGGA database. HSPA5 and MTPN are possible biomarkers of gliomas suitable for all populations to improve the prognosis of these patients.

## Introduction

Gliomas are the most common primary tumors of the central nervous system (Schneider et al., 2010; Wen & Kesari, 2008). According to the World Health Organization (WHO) classification of neurological tumors, they were classified into grade I–IV (Louis et al., 2007). The treatment mode is surgical resection combined with chemotherapy and radiotherapy (Mrugala, 2013). The prognosis of most patients is still poor due to its high incidence, high recurrence rate, high fatality rate and low cure rate. Epidemiological studies have found that the median survival time of patients with low-grade gliomas is 6 to 8 years, and that of patients with glioblastomas is only 12 to 18 months (Yang, Zhou & Lin, 2014).

With the continuous development of genome sequencing technology, a variety of new treatment methods, such as targeted therapy and immunotherapy, have been adopted in the first-line treatment. But there is no better way to treat gliomas because of their own complexity and heterogeneity that makes it difficult to obtain good biomarkers for drug development. Therefore, based on The Cancer Genome Atlas (TCGA) and the Chinese Glioma Genome Atlas (CGGA), two important genomic databases of gliomas, this study analyzes the common diagnostic and prognostic markers in Caucasian and Asian populations, which can be used as drug targets in the future.

The heterogeneity of gliomas includes histological and genetic complexity. A number of genetic tests have been included in the latest WHO classification of tumors in the central nervous system, and some genotypes and phenotypes have been included in the main diagnostic indicators (Louis et al., 2016). In light of the genetic characteristics, gliomas can be divided into Isocitrate dehydrogenase (IDH) mutation and O-6-methylguanine-DNA methyltransferase(MGMT) methylation. The research focuses involve IDH mutation, 1P/19Q combined deletion, MGMT promoter methylation, etc. Many studies have shown that IDH mutation is a common initial factor in all types of gliomas, and high-grade glioma patients with IDH mutation have a significantly better prognosis than those without mutation (Leu et al., 2016). Deletion of chromosome1P/19Q is closely related to the occurrence and development of oligodendroglioma. Previous studies have confirmed that 1P/19Q combined deletion is not only a favorable prognostic factor in oligodendroglioma, but also a marker of its sensitivity to radiotherapy and chemotherapy (Zhang et al., 2014). The occurrence and development of various malignant tumors (such as lymphoblastoma, lung cancer, esophageal cancer) are related to MGMT protein expression loss and DNA repair disorder caused by MGMT gene promoter methylation. Multiple studies have shown that glioma patients with MGMT promoter methylation are more sensitive to chemotherapy and their survival time is longer than those without methylation (Jiang et al., 2014). Although a couple of histological subtypes and molecular subtypes are already found, the prognosis is bad for some glioma patients.

Studies on protein targets mainly focus on epidermal growth factor receptor, vascular endothelial growth factor receptor, platelet-derived growth factor receptor, RAS-RAF-MEK-ERK pathway, PI3K/AKT/mTOR pathway, protein kinase C pathway, and multi-target kinase inhibitors, etc (Hide et al., 2011; Wick et al., 2011; Sathornsumetee et al., 2007; Riolfi et al., 2010; Xiao et al., 2020; Lo, 2010; Fan & Weiss, 2010; Huang et al., 2020). Molecular and genetic analysis to explore the pathogenesis of gliomas and the clinical application of targeted therapy for each subtype of gliomas is also a current research hot spot where deletion of PTEN gene, deletion or mutation of cyclin-dependent kinase inhibitor protein, etc. are studied. The increase of epidermal growth factor receptor protein is the main manifestation of primary gliomas. Hall et al. suggested that inhibition of P53 and activation of MYC signaling pathways in normal astrocytes exposed to GBM-EVs might be a mechanism by which glioblastoma(GBM) manipulates astrocytes to acquire a phenotype that promotes tumor progression (Hallal et al., 2019).

Bioinformatics methods have also been employed to find connections between RNA and gliomas. Through bioinformatics analysis, Zhang et al. (2010) found that Mir-221/222 might jointly regulate about 70 targeted genes and play a synergistic regulatory function in gliomas through the Akt signaling pathway. LncRNA including ASLNC22381 and ASLNC20819 also play important roles in the development and pathological mechanism of gliomas (Han et al., 2012). In our preceding work, we detected that increased expression of neuropilin-1 gene indicated poor prognosis for patients (Dai et al., 2017). Our team also used mRNAseq and micro (mi)RNAseq data to construct a co-expression network of gliomas and revealed the prognostic molecular signature of grade III gliomas. A total of 37 mRNAs and 10 miRNAs were identified, which were closely associated with the survival rates of patients with grade III gliomas (Bing et al., 2016). But non-coding RNAs were difficult to develop drug. The mRNA encoding protein is more suitable for drug development. Data mining of public sources of gene expression is an effective way to identify novel tumor-associated genes, and this work may contribute to the identification of candidate genes for glioma angiogenesis (Su et al., 2013).

In this study, we identified protein coding gene signature from TCGA and CGGA, and screened the top-20 differential expression genes that are related to prognosis. By literature search, we excluded the previously reported prognostic biomarkers of gliomas, and obtained two gene signatures (HSPA5 and MTPN) that can predict the prognosis of both white and Asian populations (Norris et al., 2016; Li et al., 2014; Ge et al., 2018; Yan et al., 2019). We also collected some clinical specimens to verify the expression of HSPA5 and MTPN in glioma and normal brain tissue. We hope to find biomarkers of gliomas suitable for all populations to improve the prognosis of these patients.

## Materials and Methods

### Data resource and preprocessing

In order to obtain the genes related to the prognosis of GBM patients, the phenotype information and gene expression data of tumor samples and those of normal samples were successively collected from TCGA database and The Genotype-Tissue Expression (GTEx) project, according to overall survival (OS) of glioblastoma (GBM) patients. The standardized form of TCGA data were collected from UCSC Xena (http://xena.ucsc.edu), where gene expression was recorded as the log2 transformed FPKM (Reads Per Kilobase of exon model per Million mapped reads). To validate whether the gene biomarkers were valid in Chinese people, two datasets were downloaded from CGGA (http://www.cgga.org.cn/). The following three kinds of samples were excluded: (1) patients with incomplete information of either phenotype or gene expression, (2) patients with OS time less than 30 days, (3) patients with recurrent tumor. In the TCGA database, GBM sample included 147 samples and 196 normal tissue that selected from GTEx database. In the CGGA database, three datasets were included in this database. Of these datasets, CGGA_639 and CGGA_325 were tested by RNA-Seq. Another one was performed by Affymatrix chip. So, we selected two datasets that used the same platform.

### Differential expression analysis

To identify the genes related to overall survival of GBM patients, the differentially expressed mRNAs between GBM patients and normal individuals were selected at first. The Limma package of R 3.6.1 was employed to analyze the data (https://www.r-project.org/). The threshold was set as follows: the adjusted *p* value was less than 0.001, abstract of log2-fold-change was larger than 1, B>5 and AveExpr>5. The *p* value was calculated via Student-t test and adjusted by Benjamini–Hochberg method (Puoliväli, Palva & Palva, 2020). The differentially expressed genes (DEGs) could be explained as genes expressed differently between GBM and normal tissue.

### The predictive prognostic genes

Univariate Cox (Uni-Cox) proportional hazard regression and least absolute shrinkage and selection operator (LASSO) were employed to screen the genes that are related to survival among TCGA GBM patients. Uni-Cox was applied to identify the independent effect related to overall survival of each DEG. The hazard ratio (HR) of each mRNA was calculated according to following equation:

(1) \begin{eqnarray*}\mathrm{HR}={e}^{\beta }\end{eqnarray*}

where *β* represents the coefficient from Uni-Cox. Here Survival package of R was applied.

Then to simplify the predictive genes, least absolute shrinkage and selection operator (LASSO) method was adopted, which further filtered the statistically significant mRNAs further (Zhang et al., 2019). Package glmnet of R was applied.

### Prognostic index model construction

Based on the identified prognostic genes, a risk value named prognosis index (PI) was calculated for every patient as an integrated signature. PI was calculated as follows:

(2) \begin{eqnarray*}\mathrm{PI}=\sum _{i=1}^{m}{\beta }_{i}\times {E}_{i}\end{eqnarray*}

where *β*_*i*_ represents the coefficient of the involved gene *i*, and *E*_*i*_ represents the corresponding gene’s expression level. Then normalized PI was calculated as follows:

(3) \begin{eqnarray*}\text{normalized}P{I}_{i}= \frac{P{I}_{i}-mean(PI)}{sd(PI)} \end{eqnarray*}

where *mean*(*PI*) represents the mean and *sd*(*PI*) represents the standard deviation of all PIs, respectively. The PIs below all referred to normalized PI. In order to classify the patients of high risks and low risks, the median of PIs was set as a cut-off. If the PI of a patient is larger than the cut-off, the patient is going to be assigned to high risk group, and be predicted with a bad OS, otherwise the patient will be predicted to have low risks of death.

### Analysis of clinical confounding factors with PI

Several clinical characteristics might correlate with the OS of GBM patients. In TCGA database, age, KPS score, cancer status, gender and race were considered as the main clinical confounding factors affecting prognosis (Xiong et al., 2014). Therefore, we explored the role of age, KPS score, cancer status, gender, race and PI in TCGA datasets via Uni-Cox. Following this, multivariable Cox (Multi-Cox) proportional hazard regression was also conducted to explore the joint effect of these clinical factors and the previously calculated PI, which allowed comparison of the prognostic value of PI to that of each clinical factor. In the CGGA database, clinical variables contain more information, which is helpful to understand the influence of various confounding factors on Cox regression. In the CGGA database, it mainly includes grade, gender, age, radiotherapy status, chemotherapy status, IDH mutation status and 1p19q codeletion status. The inclusion and exclusion criteria of the cases to enter into the multivariable model were listed as follows: (1) patients with clinical data were selected; (2) untreated, primary (de novo) GBM patients were selected; (3) patients with history of neoadjuvant treatment were not admissible; (4) patients more than 30-day survival were selected. Variables with *p*-values < 0.05 were selected as candidates entering into the multivariable model.

### Survival analysis and model test

To evaluate the predictive effect of PI, Kaplan–Meier survival curves were created. According to the log-rank test, the PI is considered as a good predictor if the *p* value is less than 0.05. Package Survminer of R was used.Then the time-dependent receiver operating characteristic (ROC) curve analysis was introduced to assess the model. Package survival ROC of R was applied and the area under the curve (AUC) was calculated. If AUC equals to 0.5, it indicates that the predictive effect of the model is tantamount to random allocation of patients. But if AUC is more than 0.5, it implies that its predictive effect is superior to random allocation (Kottas, Kuss & Zapf, 2014).

### Geno ontology (GO) functional enrichment

To analyze the basic biological function of genes identified in our model, package ClusterProfiler (Yu et al., 2012) of R was adopted to conduct the GO analysis of functional process and Kyoto Encyclopedia of Genes and Genomes (KEGG) pathway enrichment analysis. The *p*-value cut-off is less than 0.05 as significant enrichment threshold.

### Validation in CGGA dataset

Because most of the patients in the sample set is white, and only 8 samples are Asians (accounting for 5.5% of all samples), whether the model is valid for Chinese patients is unknown. Therefore, the validation of the model was conducted in two datasets collected from CGGA, one of which named CGGA_325 (a dataset with 325 Chinese GBM patients) and the other is CGGA_693 (a dataset with 693 Chinese GBM patients), respectively. The two datasets contain different sample numbers, among which CGGA_693 contains 693 samples, CGGA_325 contains 325 samples. The two datasets also contain different types of samples. CGGA_693 database contains primary LGG, recurrent LGG, primary GBM, recurrent GBM. CGGA_325 database contains primary LGG, recurrent LGG, primary GBM, recurrent GBM and secondary GBM. The PI of each patient was calculated according to the mRNAs identified in the model, and then K-M curves and ROC curves analysis were performed. Meanwhile, several clinical factors recorded in CGGA datasets were added into multivariable COX model to validate the predictive effects of PI when adjusted for confounders.

### Human tissue samples

A total of 30 glioma tissue specimens and 10 cases of peritumor brain tissue (used control) resected by corticostomy were collected from Lanzhou University Second Hospital between October 2019 and May 2020. All these patients did not receive preoperative radiotherapy, chemotherapy or other immunotherapy, and were confirmed by surgical pathology. According to the WHO classification, there were 15 cases of WHO Grade II. Among them, 9 cases are of males, 6 cases are of females and the age ranges from 21 to 59 (40.5 ± 9.6). There were 9 cases with diffuse astrocytoma (A), 4 cases with oligodendroglioma (O), 2 cases with oligoastrocytoma (OA). There were 15 cases of WHO Grade III/IV, among which 6 cases are of males, 9 cases are of females, and the age ranges from 29 to 69 (47.9 ± 12.9). There were 4 cases with Anaplastic astrocytoma (AA), 1 case with anaplastic oligodendroglioma (AO), 4 cases with anaplastic oligoastrocytoma (AOA), 6 cases with glioblastoma (GBM). Among control groups, 5 cases are of males, 5 cases are of females, and the age ranges from 31 to 69 (49.7 ± 11.4). This study was approved by the Ethics Committee of Lanzhou University Second Hospital (2020A-147), and all patients signed informed consent before surgery.

### RNA extraction and qRT-PCR

Total RNA from all cells and tumor tissues were isolated using Trizol reagent (Takara, Dalian, China) and the first-strand cDNA was converted using the PrimeScript RT reagent Kit with genomic DNA Eraser (Takara). Then, TB Green Premix ExTaq (Takara) was used to perform qRT-PCR (Bio-Rad CFX96). Based on the results of differential analysis, GAPDH was selected as reference gene in glioma samples. Relative gene expression was evaluated by a comparative CT method (2-ΔΔCtmethod). Statistical significance of qRT-PCR data was analyzed using IBM SPSS Statistics 22.0 software(Armonk, NY, USA) and determined by the Student’s *t*-test. *p* < 0.05 was considered to be statistically significant. Primers used were as following: HSPA5 forward: 5′-GACATCAAGTTCTTGCCGTTCA-3′, HSPA5 reverse: 5′-CCAGCAATAGTTCCAGCG TCTT-3′; MTPN forward: 5′-CGGAGACTTGGATGAGGTGAA-3′, MTPN reverse: 5′-AGAGCTTTGATTGCCTGGTTG-3′; GAPDH forward: 5′-GGAAGCTTGTCATCAAT GGAAATC-3′, GAPDH reverse: 5′-TGATGACCCTTTTGGCTCCC-3′.

### Western blot assay

Tissue samples were lysed in RIPA lysis buffer, and lysates were harvested by centrifugation (12,000 rpm) at 4 °C for 30 min. Western blot was performed in accordance with the protocols as described above, using *β*-actin as the internal control. Briefly, the protein sample was separated by SDS-PAGE electrophoresis and then transferred to a PVDF membrane. After blocking nonspecific binding sites for 60 min with 5% non-fat milk, the membrane was incubated with the primary antibody at 4 °C overnight. Membranes were washed three times with tris buffered saline with 1‰tween-20 and incubated with horseradish peroxidase-conjugated secondary antibody at 37 °C for 1 h. After 3 washes, the bands were detected by an enhanced chemiluminescence system (WBKLS0500, Merck KGaA). Band density was measured using ImageJ software (National Institutes of Health, Bethesda, MD) and standardized to that of *β*-actin. Antibodies against proteins and dilution multiples are as follows: HSPA5(ab21685, abcam, USA) (1:1000), MTPN(bs-11891R,bioss,China) (1:1000), *β*-actin (service, China)(1:3000).

### Immunohistochemical assay

Two-Step method was adopted to detect the expression of HSPA5 and MTPN. Tissue specimens were fixed promptly with 100g/L formaldehyde solution, embedded in paraffin and cut into 3 µm sections. All sections were dehydrated with graded alcohol, repaired with antigen, quenched with peroxidase solution, put into tap water, and washed with PBS. The slides were incubated in a moist chamber with HSPA5 or MTPN rabbit polyclonal antibody (1:100) at 37 °C for 30min. Then they were incubated in a moist chamber with the goat polyclonal antibody against rabbit at 37 °C for 30min. After being washed in PBS completely, the slides were developed in 0.05% freshly prepared diaminobenzedine solution (DAB) for 10 min, and then counterstained with hematoxylin. Finally, the slides were dehydrated in ascending concentrations of ethanol, airdried, and mounted. The expression of HSPA5 and MTPN were scored according to the degree of staining and the number of stained cells. Degree of staining: 0 for non-staining, 1 for light yellow, 2 for brownish, and 3 for tan. Stained cell counts are defined as follows: Under high power microscope, 5 fields were randomly selected from each section to count the percentage of stained cells; 0 point is given to match the stained cells which represent less than 5%; 1 point matches 5% to 25%; 2 points matches 26%∼50%; 3 points matches 51%∼75%; 4 points matches more than 75%. The product of the two scores is scored:0 is negative, 1-3 is weakly positive, 4-5 is moderately positive, and ≥6 is strongly positive. Negative and weak positive were classified as negative expression, while moderate positive and strong positive were classified as positive expression.

### Statistical analysis

Statistical analysis of human tissue data was expressed as mean ± SD (standard deviation) from three independent experiments. Differences between groups were estimated using the Student’s *t*-test. Discrete data values were expressed as rate and analyzed by chi-squared test. All these analyses were performed using IBM SPSS Statistics 22.0 software (Armonk, NY, USA) and a two-tailed value of *P* < 0.05 was considered statistically significant.

## Results

### DEGs Between GBM samples and Non-GBM samples

By preprocessing, 147 GBM patients with 19,199 genes in TCGA and 196 non-GBM individuals with 19199 genes in GTEx were collected to do the following analysis.

**Figure 1:**
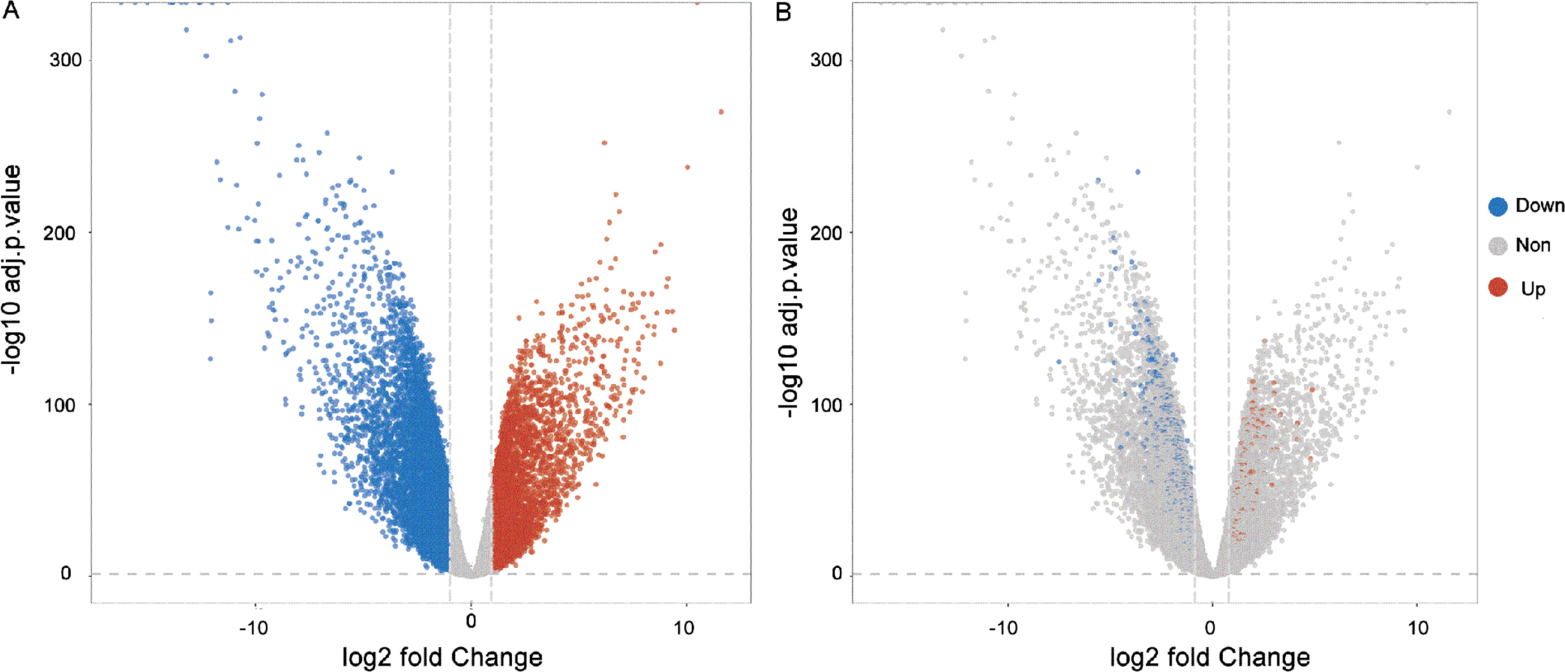
Volcano plots of the DEGs. (A) Blue dots implied the genes were down-regulated and red dots were up-regulated. The grey plots implied the mRNAs were not differentially expressed significantly. (B) Blue and red dots indicate genes with an average expression of more than 5.

Through the analysis of differential gene expressions, 581 DEGs were left in the model according to the threshold. Among them, 138 mRNAs were highly expressed and 443 mRNAs were lowly expressed. Figure 1 shows the volcano plots of the results of DEGs. For further biological experiment, we filtered average expression more than 5 as threshold. The gene significantly expression showed in Fig. 1A. Filtering genes were showed in Fig. 1B.

### The survival related genes of GBM

Through Uni-cox regression, 50 mRNAs were identified as survival related genes according to *p* value less than 0.05 in TCGA database. Then LASSO method was employed and 20 mRNAs were finally selected. The information of these 20 genes were listed in Table 1. Among them, 16 mRNAs were high-risk genes with HRs from 1.230 to 2.039, which meant that the higher their expression, the worse OS the patient might have. In contrast, 4 genes were protective factors with HRs from 0.789 to 0.552.

**Table 1:** Survival related genes (TCGA).

Genes	Coefficients of Uni-Cox	HR	95%CI for HR	*p*-value
**High Risk Genes**				
RNF10	0.713	2.039	1.013–4.105	0.046^*^
MTPN	0.593	1.809	1.141–2.868	0.012^*^
RTN4	0.540	1.717	1.078–2.734	0.023^*^
HSPA5	0.518	1.679	1.175–2.398	0.004^**^
PLD3	0.511	1.667	1.134–2.451	0.009^**^
GRN	0.495	1.640	1.176–2.287	0.004^**^
FLII	0.467	1.595	1.068–2.383	0.023^*^
NDUFB2	0.397	1.488	1.026–2.158	0.036^*^
DKK3	0.365	1.441	1.133–1.831	0.003^**^
MAP1LC3A	0.361	1.435	1.130–1.822	0.003^**^
SERPINE2	0.329	1.390	1.079–1.791	0.011^*^
TTYH3	0.285	1.330	1.056–1.674	0.015^*^
SCG5	0.258	1.294	1.037–1.615	0.023^*^
FN1	0.250	1.284	1.047–1.576	0.017^*^
TAGLN2	0.239	1.270	1.051–1.534	0.013^*^
LY6E	0.207	1.230	1.017–1.489	0.033^*^
**Low Risk Genes**				
RPS19	−0.241	0.786	0.620–0.996	0.046^*^
EIF3L	−0.441	0.643	0.469–0.883	0.006^**^
EIF4A2	−0.447	0.639	0.430–0.951	0.027^*^
FDPS	−0.594	0.552	0.333–0.916	0.021^*^
**Integrated Genes**				
PI	0.976	2.653	1.780–3.955	<0.001

**Notes.**

* *p* < 0.05.

** *p* < 0.01.

### PI construction

**Figure 2:**
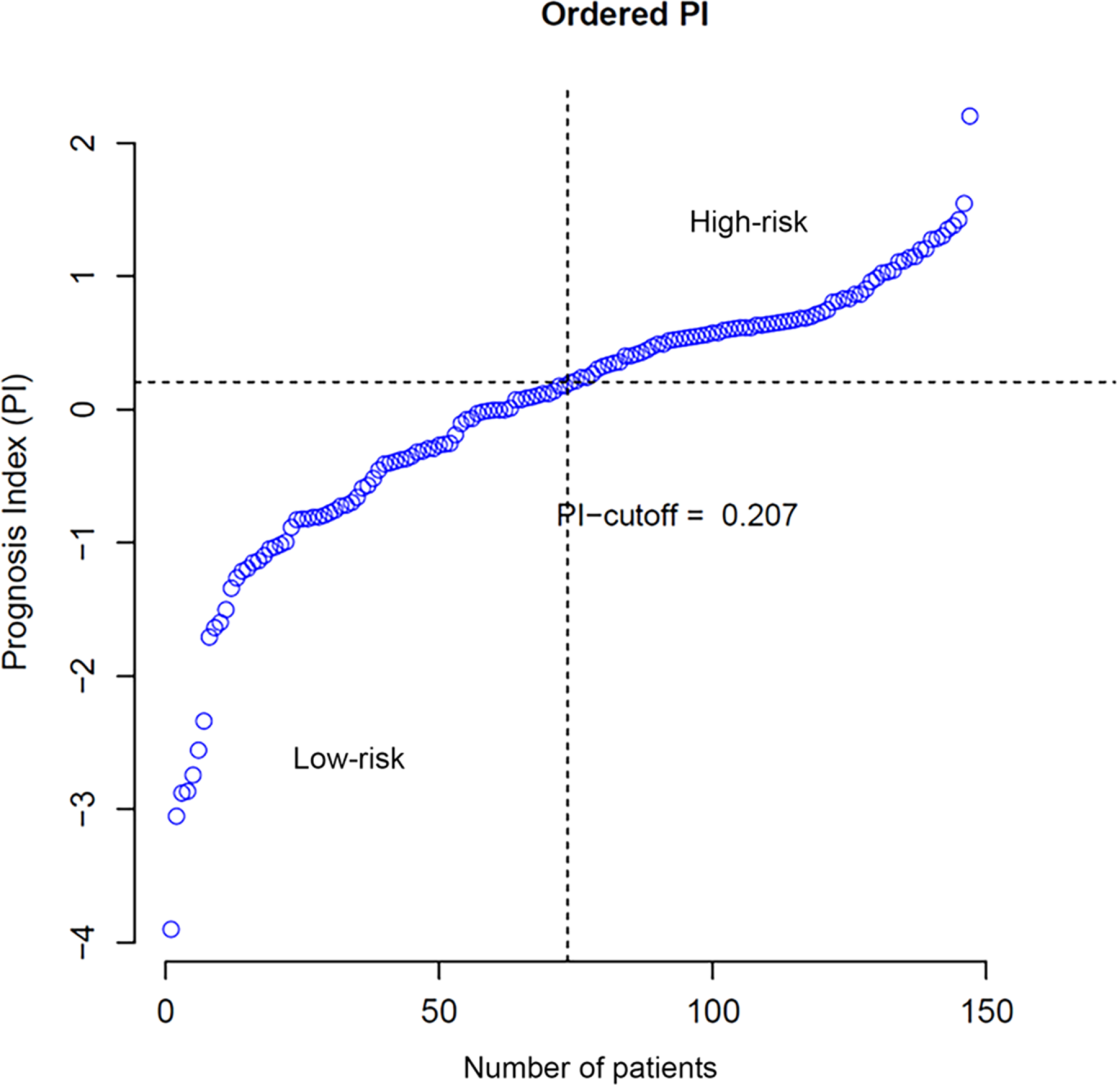
Distribution of patients according to PI value.

According to Eqs. (2) and (3), the PI of each patient was calculated and the cut-off was set to 0.207, which implied patients with PIs larger than 0.207 were considered with high risks of overall survival. Ordered PIs were depicted in Fig. 2. The HR of PI, which was displayed in the last line of Table 1, was 2.653 with 95% CI from 1.976 to 4.122. The *p*-value of Uni-Cox of PI was less than 0.001.

### Clinical factors and their joint effect with PI

Table 2 summarizes the results of clinical factors analysis. Five clinical factors were included in the analysis according to the characteristics of the original training set. They are age, karnofsky performance score (KPS), cancer status, gender and race of patients. Based on Uni-cox and survival analysis, it showed that age, KPS and cancer status were independent significant factors to OS of GBM patients. Then, through multivariate analysis, only KPS, cancer status and PI were statistically significant. HR of PI is 2.653 (95% CI [1.780–3.955], *p* < 0.001), which demonstrated PI is significant risk factor.

**Table 2:** Cox hazard regression of clinical factors and PI.

Factors	Uni-Cox					Multi-Cox
	Numbers	Coef	HR	95%CI for HR	*p*	*p*
Age (>= 45 VS <45)	128/19	0.982	2.669	1.382–5.155	0.004^**^	0.202
KPS (>= 50 VS <50)	105/7	−1.606	0.201	0.077–0.523	0.001^**^	0.001^**^
Cancer status (WITH TUMOR VS TUMOR FREE)	118/12	1.351	3.860	1.535–9.707	0.004^**^	0.049^*^
Gender (male VS female)	95/52	−0.091	0.913	0.624–1.338	0.641	0.555
Race (white VS not white)	133/13	−0.090	0.914	0.444–1.882	0.808	0.375
PI (high-risk VS low-risk)	73/74	0.976	2.653	1.780–3.955	<0.001	<0.001

**Notes.**

* *p* < 0.05.

** *p* < 0.01.

### Survival analysis and model test

**Figure 3:**
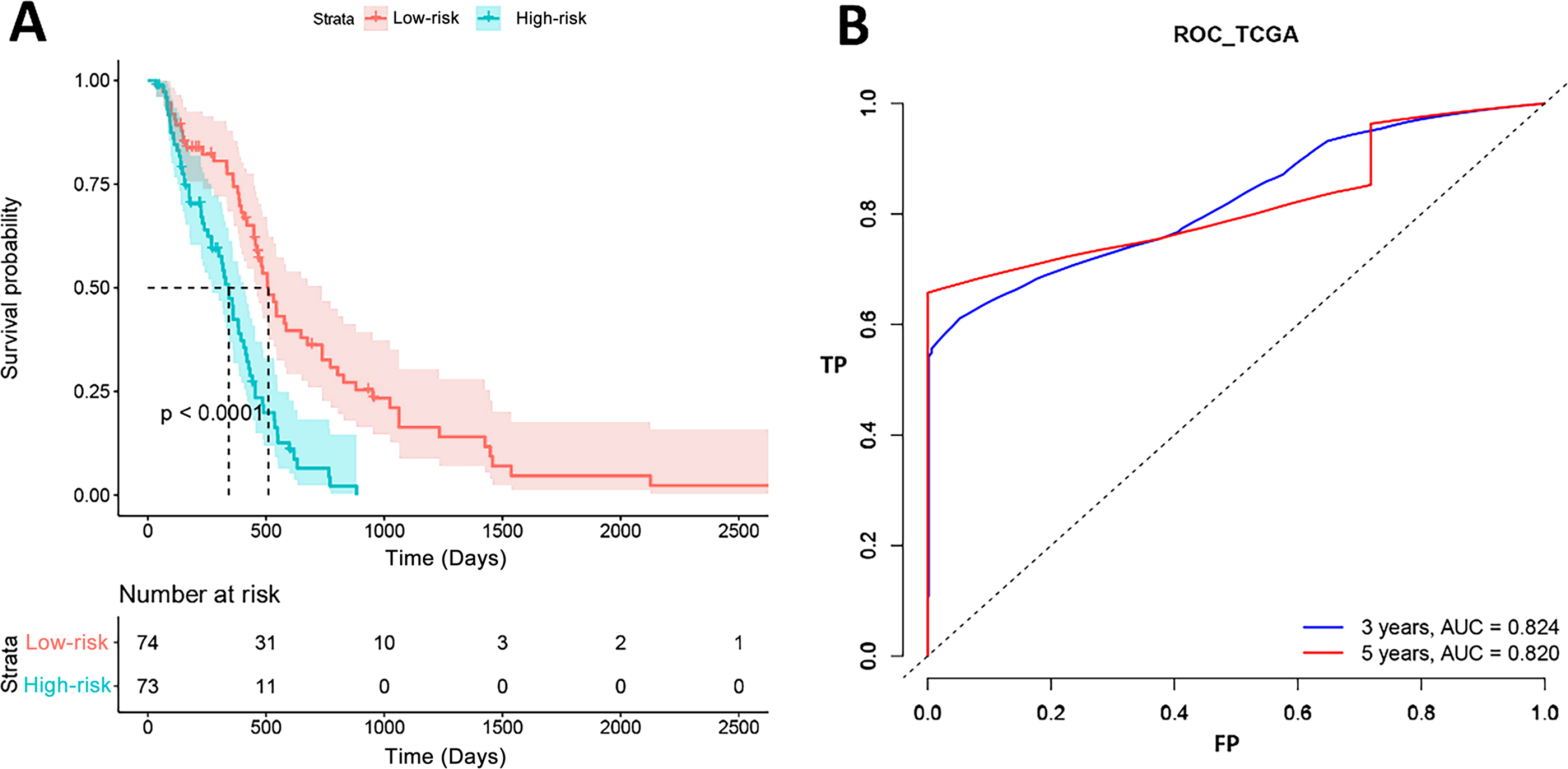
Survival analysis results in TCGA. (A) High-risk and low-risk groups showed significantly different survival. (B) ROC curve showed the performance of PI in TCGA.

Figure 3 shows the results of K-M curves of PI. The red line was the survival curve of the low-risk patients and the green line was that of high-risk patients. For 500 days, 31 patients (41%, 31/74) were alive in the low-risk group and 11 patients (15%, 11/73) in the high-risk group. For 1,000 days, all patients in the high-risk groups were dead, while 10 patients (13%, 10/74) in the low-risk group were still alive. And the *p* value of log-rank test was less than 0.001. The right term of the figure was the ROC curves of our model. The AUCs of 3 years and 5 years were 0.824 and 0.820, respectively, which represented that our model had a good prognostic effect for GBM.

### Results of GO enrichment analysis

**Figure 4:**
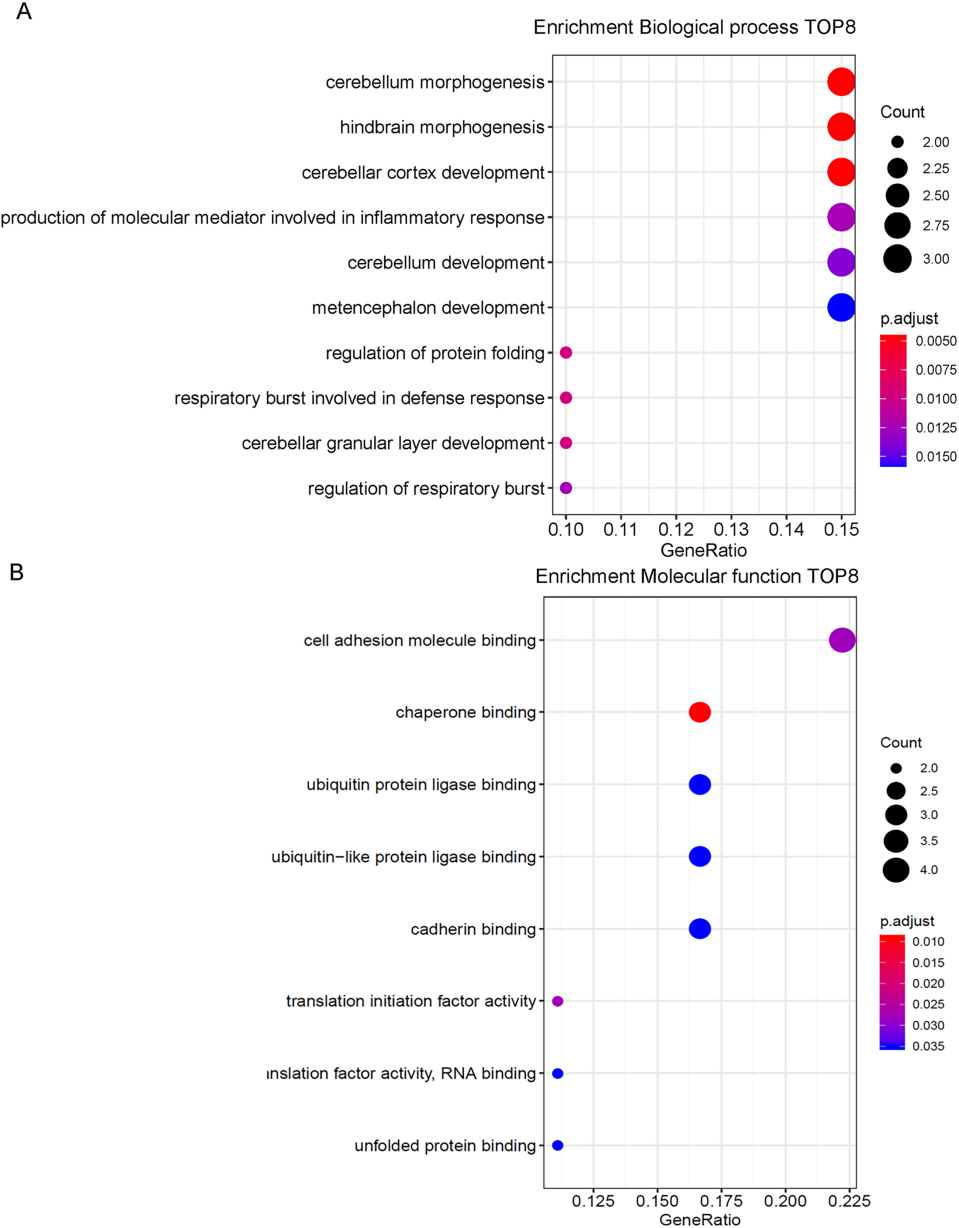
GO enrichment analysis. (A) Top eight genes involved in biological process. (B) Top eight genes involved in molecular function.

According to GO functional enrichment analysis, 8 biological processes were enriched with adjusted *p* value less than 0.05 (Fig. 4). They were cell adhesion molecule binding, chaperone binding, ubiquitin protein ligase binding, ubiquitin-like protein ligase binding, cadherin binding, translation initiation factor activity, translation initiation factor activity, translation factor activity-RNA binding and unfolded protein binding. DEGs mainly involved in cell adhesion molecule binding, chaperone binding, ubiquitin protein ligase binding, ubiquitin-like protein ligase binding and cadherin binding. These genes are engaged in the biological processes of various brain functions such as cerebellum morphogenesis, hindbrain morphogenesis, cerebellar cortex development and etc. In molecular function, these genes play a part in binding function.

### Validation in CGGA dataset

In validation section, we applied above results from TCGA to test in CGGA datasets. The Uni-Cox results of 20 mRNAs were listed in Table 3. It showed us that among 20 identified genes through TCGA, 4 genes (MTPN, RTN4, MAP1LC3A and DKK3) were significantly associated with OS. Although MTPN is not remarkably associated with OS, the results of calculation showed that HR>1 of MTPN and *p*-value showed a significant trend.

**Table 3:** Uni-Cox results in CGGA datasets.

	CGGA_693				CGGA_325			
Genes	Coef	HR	95%CI for HR	*p*	Coef	HR	95%CI for HR	*P*
TTYH3	0.452	1.571	1.391–1.775	<0.001	0.729	2.072	1.743–2.464	<0.001
TAGLN2	0.423	1.526	1.406–1.656	<0.001	0.652	1.919	1.707–2.158	<0.001
FN1	0.336	1.399	1.301–1.504	<0.001	0.484	1.623	1.479–1.781	<0.001
HSPA5	0.235	1.265	1.113–1.438	<0.001	0.783	2.188	1.843–2.597	<0.001
EIF3L	−0.156	0.856	0.810–0.904	<0.001	−0.772	0.462	0.358–0.597	<0.001
SCG5	−0.088	0.916	0.863–0.972	0.004^**^	−0.194	0.823	0.739–0.917	<0.001
SERPINE2	−0.128	0.880	0.806–0.961	0.005^**^	−0.691	0.501	0.395–0.635	<0.001
LY6E	−0.136	0.873	0.802–0.949	0.001^**^	0.027	1.028	0.841–1.257	0.789
FDPS	−0.158	0.854	0.775–0.941	0.001^**^	−0.569	0.566	0.365–0.877	0.011^*^
GRN	0.135	1.145	1.018–1.287	0.023^*^	0.930	2.534	2.116–3.034	<0.001
FLII	0.127	1.136	0.997–1.294	0.056	1.463	4.318	3.239–5.756	<0.001
MTPN	0.102	1.107	0.959–1.279	0.166	0.148	1.160	0.858–1.568	0.335
PLD3	0.059	1.061	0.928–1.213	0.388	0.480	1.615	1.294–2.017	<0.001
EIF4A2	−0.002	0.998	0.913–1.091	0.967	−0.557	0.573	0.412–0.798	0.001^**^
RTN4	−0.014	0.986	0.870–1.117	0.820	−0.240	0.787	0.537–1.153	0.219
RNF10	−0.033	0.968	0.850–1.103	0.624	1.123	3.075	2.253–4.198	<0.001
RPS19	−0.034	0.966	0.915–1.020	0.218	0.850	2.340	1.852–2.955	<0.001
NDUFB2	−0.046	0.955	0.907–1.005	0.078	0.543	1.721	1.282–2.309	<0.001
MAP1LC3A	−0.049	0.953	0.880–1.031	0.228	−0.009	0.991	0.851–1.154	0.912
DKK3	−0.074	0.929	0.822–1.049	0.235	−0.015	0.985	0.832–1.165	0.859
**Integrated Genes**								
PI	1.649	5.200	3.872–6.983	<0.001	1.875	6.524	4.496–9.466	<0.001

**Notes.**

* *p* < 0.05.

** *p* < 0.01.

Seven of these genes only were statistically significant via Uni-Cox in either CGGA_693 or CGGA_325 (LY6E, FLII, PLD3, EIF4A2, RNF10, RPS19 and NDUFB2). The other 9 genes (TTYH3, TAGLN2, FN1, HSPA5, EI3L, SCG5, SERPINE2, FDPS and GRN) were still prognostic genes in both CGGA_693 and CGGA_325. Among these nine genes, SCG5 and SERPINE2 showed the opposite effects in CGGA and TCGA, which might because of racial difference. And the seven genes mentioned above showed the same effects (protective factor or risk factor) between TCGA and CGGA, which might be the real prognostic genes of GBM. The HRs of PI in CGGA_693 and CGGA_325 were 5.200 (95%CI [3.872–6.983]) and 6.524 (95%CI [4.496–9.466]), respectively. Thus our 20-gene model still had a good predictive effect among Chinese GBM patients. As is shown in Table 4, WHO clinical grade and 1p19q codelection status were also significant high-risk clinical factors of GBM patients for overall survival. Accounting for clinical confounders, PI was still a critical prognostic predictor in both CGGA_693 and CGGA_325 datasets.

**Table 4:** Validation in CGGA datasets adjusted for clinical factors.

	CGGA_693				CGGA_325			
	Coef	HR	95%CI for HR	*P*	Coef	HR	95%CI for HR	*P*
PI (high-risk VS low-risk)	0.884	2.422	1.577–3.718	<0.001	0.667	1.948	1.185–3.202	0.009^**^
Grade (WHO III VS WHO II)	1.260	3.524	2.091–5.939	<0.001	1.129	3.094	1.716–5.580	<0.001
Grade (WHO IV VS WHO II)	1.731	5.648	3.097–10.301	<0.001	1.380	3.974	2.150–7.348	<0.001
Gender (Male VS Female)	−0.009	0.991	0.702–1.399	0.960	−0.064	0.938	0.643–1.370	0.742
Age (>= 45 VS <45)	0.385	1.469	1.025–2.107	0.036^*^	0.203	1.224	0.805–1.862	0.344
Radio_status (Radio_therapy VS Non-Radio_therapy)	−0.659	0.517	0.305–0.877	0.014^*^	−0.102	0.903	0.489–1.667	0.745
Chemo_status (Chemo_therapy VS Non-Chemo_therapy)	−0.186	0.830	0.518–1.330	0.439	−0.359	0.698	0.452–1.079	0.106
IDH_mutation_status (Wildtype VS mutant)	0.336	1.400	0.897–2.184	0.139	0.143	1.153	0.703–1.893	0.573
1p19q_codeletion_status (Non-code VS Codel)	1.081	2.947	1.510–5.751	0.002^**^	1.491	4.442	2.106–9.369	<0.001

**Notes.**

* *p* < 0.05.

** *p* < 0.01.

### Verification gene signature performance of CGGA dataset

**Figure 5:**
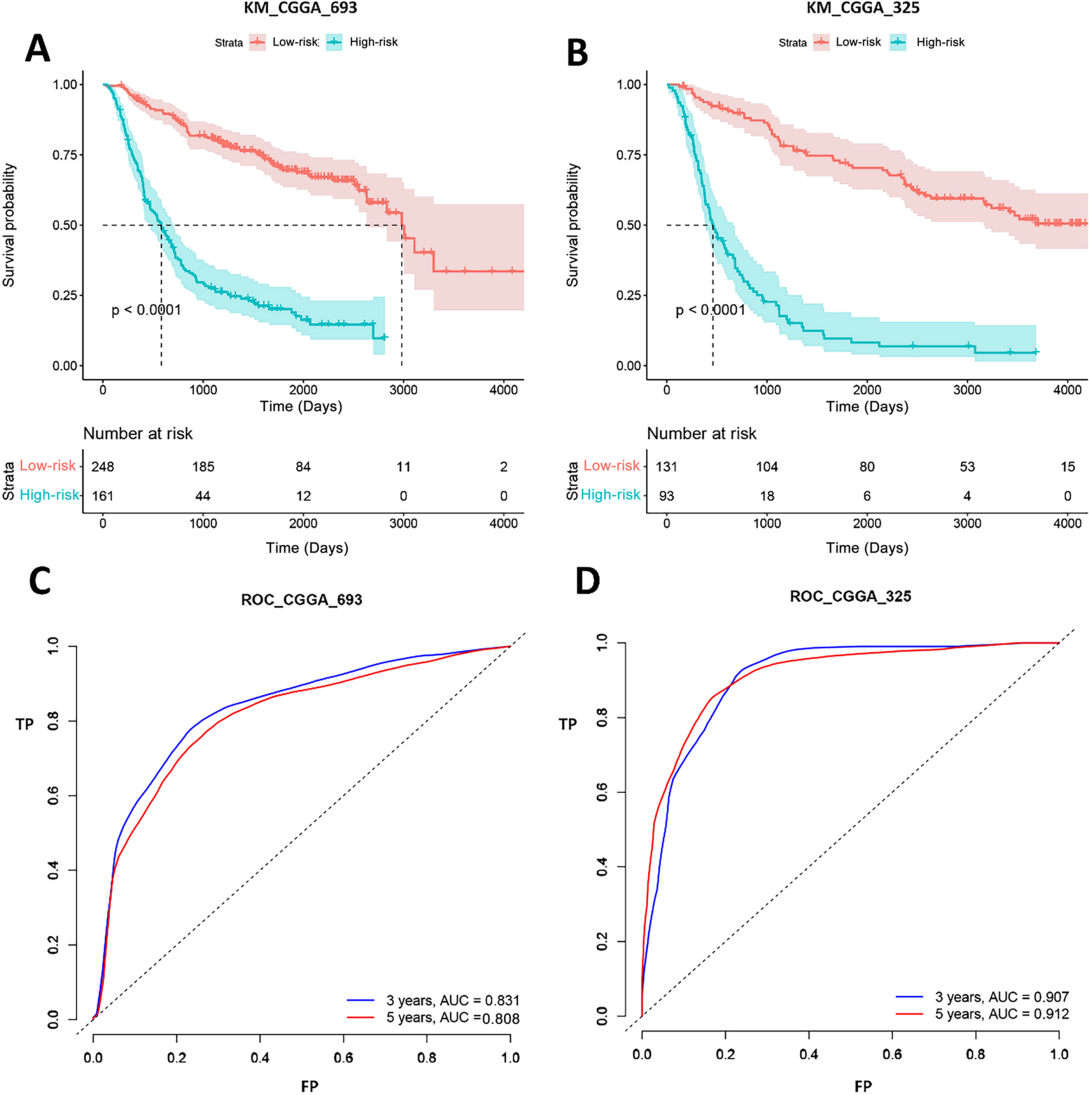
K-M curves and ROC analysis of our signature in CGGA datasets. (A) and (B) shows the K-M curves of our signature in CGGA_693 and CGGA_325 datasets, respectively; (C) and (D) shows the ROC curves of our signature in CGGA_693 and CGGA_325 datasets, respectively.

Gene signature performance was analyzed in two CGGA datasets. Survival curve and ROC curve were employed to test prediction performance of the gene signature. Figure 5 showed that the *p*-values of log-rank test of K-M curves were less than 0.0001 both in CGGA_693 and CGGA_325. In CGGA_693, 185 patients (74%, 185/248) were alive in the low-risk group and 44 patients (27%, 44/161) in the high-risk group for 1,000 days. And all patients in the high-risk group were dead, while 11 patients (4%, 11/248) in the low-risk group were still alive for 3,000 days. In CGGA_325, 104 patients (79%, 104/131) were alive in the low-risk group and 18 patients (19%, 18/93) in the high-risk group for 1,000 days. And all patients in the high-risk group were dead, while 15 patients (11%, 15/131) in the low-risk group were still alive for 4,000 days. This implied that the biomarkers could efficiently classify the Chinese GBM patients into good and poor prognosis groups. ROC analysis represented that the AUCs of CGGA_693 were 0.831 and 0.808 for 3 and 5 years, respectively, and AUCs of CGGA_325 were 0.907 and 0.912 for 3 and 5 years, respectively. Therefore, our forecasting model for GBM had a good classificaion ability in CGGA datasets. To test the predictive effects of our 20-gene model among Chinese GBM patients further, K-M curves and ROC analysis were conducted.

### The expression of mRNA HSPA5 and MTPN by PCR

**Figure 6:**
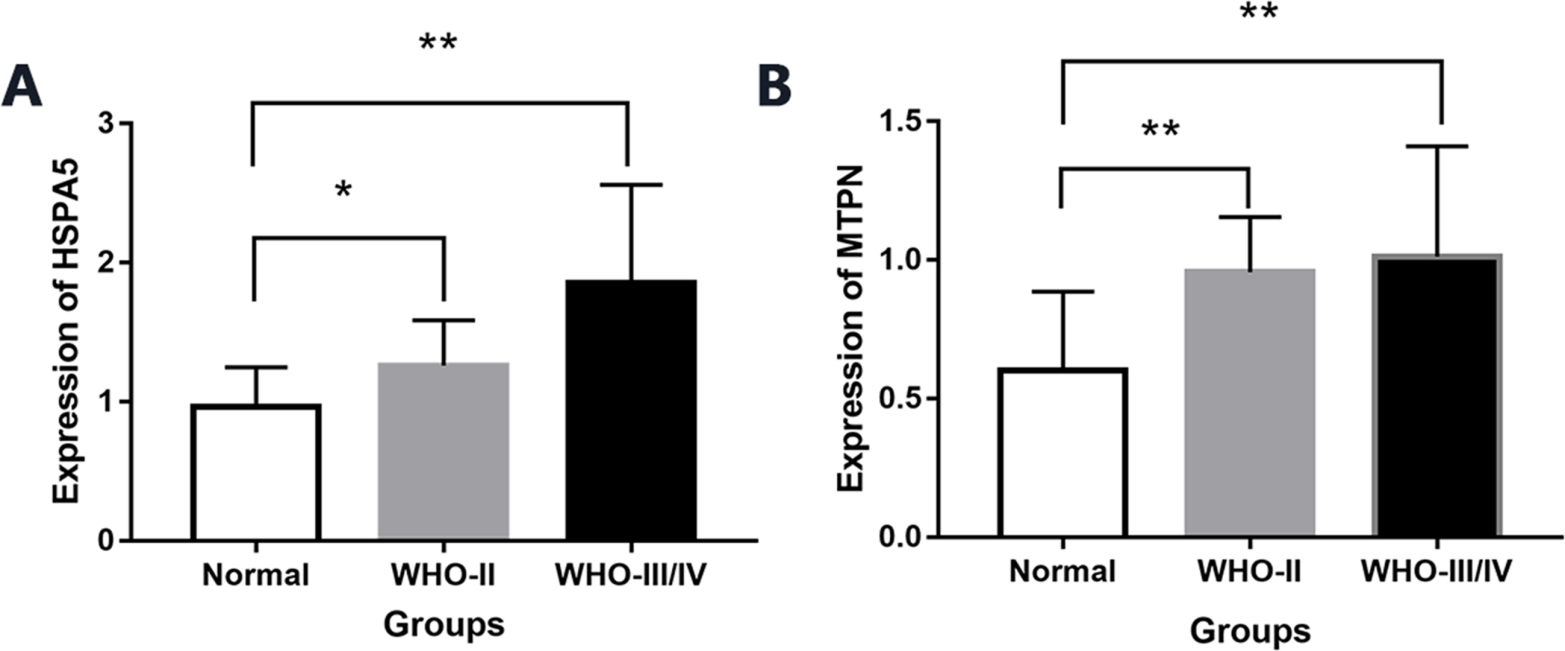
The expression of mRNA HSPA5 and MTPN. (A) The expression of mRNA HSPA5. (B) The expression of mRNA MTPN *represents *p* < 0.05 and **represents *p* < 0.01.

By real-time quantitative PCR detection, compared with normal brain tissue (0.96 ± 0.28), the expression of mRNA HSPA5 in WHO II gliomas and WHO III/IV glioma tissue both increased significantly (1.25 ± 0.32,1.85 ± 0.70), and the differences were statistically significant (*P* < 0.05, Fig. 6).

Compared with normal brain tissue (0.60 ± 0.28), the expression of mRNA MTPN in WHO II gliomas and WHO III/IV glioma tissues also increased greatly (0.95 ± 0.19,1.01 ± 0.39), and the difference was statistically significant (*P* < 0.05, Fig. 6).

We filtered all gene signature in three datasets. All genes were significantly changed in three datasets. These genes included six genes (TAGLN2, HSPA5, FN1, TTYH3, GRN and MTPN). Of these genes, TAGLN2 (Beyer et al., 2018; Han et al., 2017), FN1 (Yu et al., 2020; Gu, Gu & Shou, 2014; Guo, Heller & Thorslund, 2016; Liao et al., 2018), TTYH3 (Weinberg et al., 2020), GRN, (Ness, Riemenschneider & Baches., 2009; Trigos et al., 2019) have been reported in GBM.

### Western blot analysis

**Figure 7:**
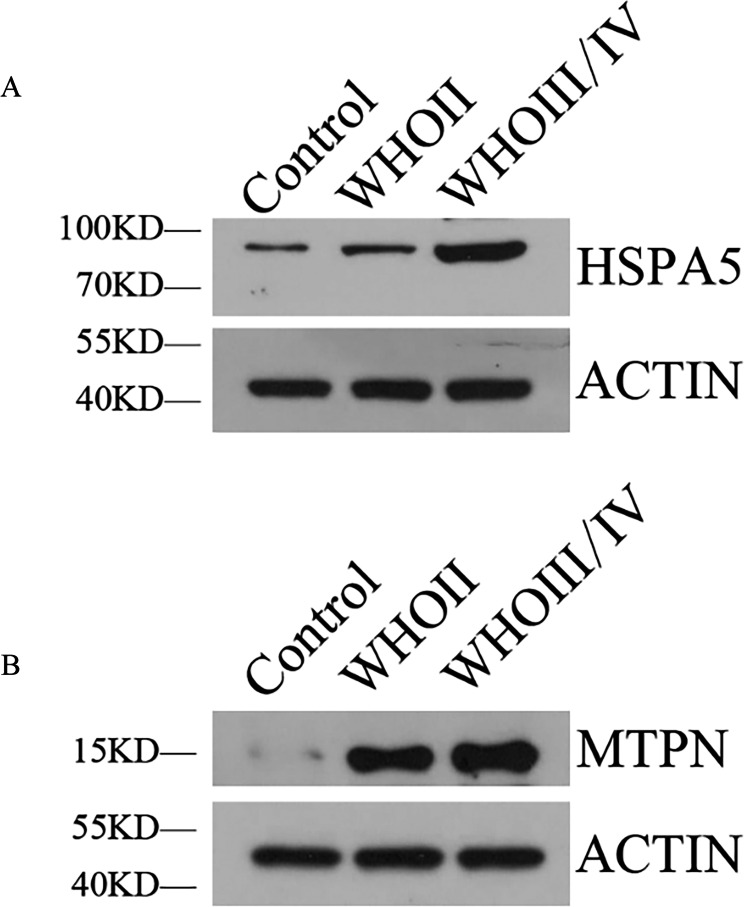
The expression of HSPA5 and MTPN by Western blot. (A) The expression of HSPA5 protein. (B) The expression of MTPN protein.

Western blotting showed HSPA5 protein level in WHO II glioma tissue and WHO III/IV glioma tissue were both elevated obviously compared with normal brain tissue. MTPN protein expression in WHO II glioma tissue and WHO III/IV glioma tissue were also significantly higher than normal brain tissue (Fig. 7).

### Immunohistochemical analysis

HSPA5 protein was mainly expressed in the cytoplasm while MTPN protein was expressed in the nucleus and cytoplasm.

**Figure 8:**
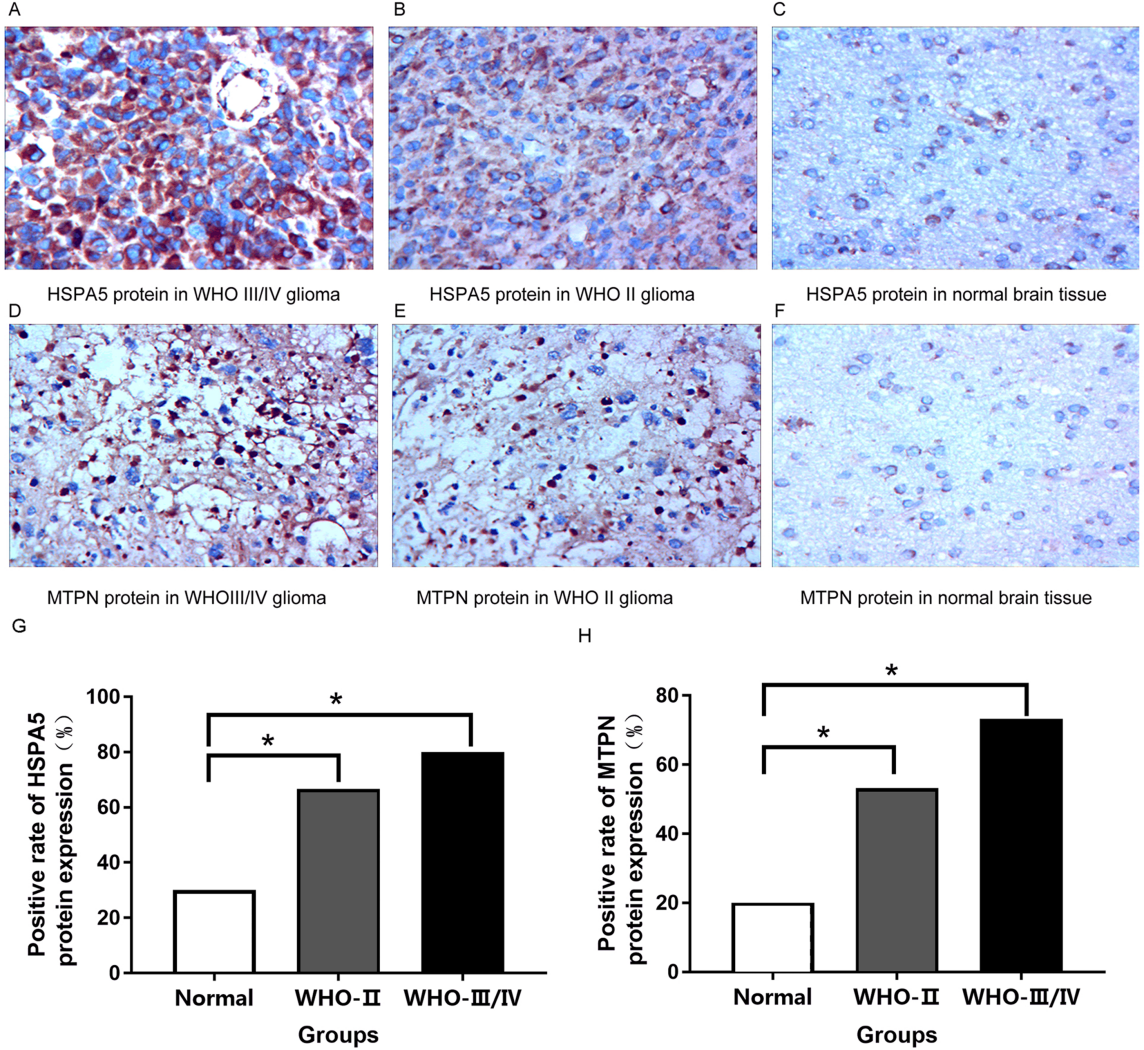
Immunohistochemical analysis in normal brain tissue and glioma (400×). (A) Strongly positive HSPA5 expression; (B) Moderately positive HSPA5 expression; (C) Negative HSPA5 expression; (D) Strongly positive MTPN expression; (E) Moderately positive MTPN expression; (F) Negative MTPN expression; (G) the positive rate of HSPA5 protein expression (%); (H) the positive rate of MTPN protein expression (%) *represents *p* < 0.05.

Immunohistochemical results indicated that compared with normal brain tissue, the positive expression of HSPA5 protein in gliomas rose obviously. For the most part, the expression of protein was strongly positive in grade III/IV glioma tissue, moderately positive in grade II glioma tissue, and mostly negative or weakly positive in normal brain tissue (Figs. 8A–8C, 8G). It is equally true of the expression of MTPN protein in gliomas (Figs. 8D–8F, 8H).

## Discussion

In recent years, the relationship between some genes or signal pathways and the occurrence and development of gliomas has been discoverd by bioinformatics analysis. In this study, many prognostic genes of gliomas have been identified from TCGA and CGGA databases by data mining and biological experiment validation. Of these genes, many biomarkers have been identified in previous studies. Relevant teams analyzed the glioma data obtained from TCGA and verified the expression of differential proteins in the tissues or body fluids of glioma patients, animal models and cell lines. Common targets are GFAP (Wang et al., 2018), HSP70 (Sharifzad et al., 2020), VEGF (Nicolas et al., 2019), BDNF (Huo & Chen, 2019), ECM (Tejero et al., 2019), FN1 (Yu et al., 2020; Gu, Gu & Shou, 2014; Guo, Heller & Thorslund, 2016; Liao et al., 2018), CD44 (Mooney et al., 2016), Fransgelin-2, Short Hairpin RNAGLN and GRN (Ness, Riemenschneider & Baches., 2009; Trigos et al., 2019) etc. Previous research suggested that TAGLN2 might be involved in progression due to its higher expression in glioblastomas compared to IDH1/2 WT gliomas of lower grades (Beyer et al., 2018; Han et al., 2017). A recent study showed that FN1 gene expression was higher in glioma tissues than in normal tissues. GO enrichment analysis and KEGG pathway enrichment analysis indicated that FN1 was involved in the synthesis of extracellular matrix (ECM) components and the PI3K/AKT signaling pathway. It was found that FN1 gene could inhibit cell proliferation, promote cell apoptosis and senescence, and reduce migration and invasion through the down-regulation of FN1 gene expression and disruption of the PI3K-AKT signaling pathway (Liao et al., 2018). Long, H.et al. demonstrated the importance of some genes, such as COL3A1, FN1, and MMP9 for glioblastoma. Based on the selected genes, a prediction model was built and its predictive accuracy was found to be 94.4%. These findings might provide more insights into the genetic basis of glioblastoma (Long et al., 2017).

In this study, we excluded the genes that have been reported and expressed the opposite effects in CGGA and TGGA, instead we focus HSPA5 and MTPN as important gene signature in both TCGA and CGGA databases. HSPA5 is GRP78 (Glucoregulated Protein 78), belonging to the heat shock Protein 70 family, usually located in the endoplasmic reticulum. The role of this gene is to maintain the biological process of endoplasmic reticulum and homeostasis. It was reported that this gene could protect organs and tissues from pathological damage. HSPA5 can regulate endoplasmic reticulum stress, initiate unfolded protein response (UPR), and improve cell viability in case of hypoxia, low glucose, and other stress states. Moreover, it is not confined to the endoplasmic reticulum of tumor cells. It can migrate to cell membranes, cell fluids, mitochondria, and nucleus, and can even be secreted. It was reported that this gene is over-expressed in many types of cancers however the high expression of HSPA5 in glioma was seldom reported. The results of the study showed that the gene expression level of HSPA5 increased with the grades of gliomas. According to data mining and patient tissue analysis, the expression of HSPA5 is positively correlated with the malignant degree of tumor. In addition, corresponding peptide drugs would be developed accordingly. Previous studies screened a novel peptide sequence SNTRVAP (VAP for short) with high binding affinity to HSPA5 in vitro and in vivo using phage display technology.

MTPN can promote dimerization of NF-kappa-B subunits and regulates NF-kappa-B transcription factor activity. This gene plays a role in the regulation of the growth of actin filaments. MTPN has been reported in breast cancer (Muñiz Lino et al., 2014). However, it has never been reported in gliomas. Bioinformatics analysis and biochemical tests showed that the gene was also highly expressed in high-grade gliomas, but low in low-grade gliomas and normal brain tissues.

In this study, we established reliable tumor markers through data analysis combined with biological experiments to provide scientific basis for future drug development.

## Conclusions

Through analysis of differential gene expressions, 581 DEGs were left according to our thresholds. Among them, 138 mRNAs were highly expressed and 443 mRNAs with low expression levels, 20 mRNAs were identified as survival related genes. The 20-gene signature can forecast the risk of Glioma in TCGA effectively, moreover it can also predict the risks of Chinese patients through validation in the CGGA database. Our study suggests that HSPA5 and MTPN are possible biomarkers of gliomas suitable for all populations to improve the prognosis of these patients.

##  Supplemental Information

10.7717/peerj.11350/supp-1 Supplemental Information 1 Raw data

## Additional Information and Declarations

### Data Availability

The following information was supplied regarding data availability: The raw measurements are available in the Supplemental File.
