# Supplementary figures and images for: Identification of robust diagnostic and prognostic gene signatures in different grades of gliomas: a retrospective study
Source: PeerJ. 2021 May 11;9:e11350. doi: 10.7717/peerj.11350 (PMC8121073; doi:10.7717/peerj.11350)

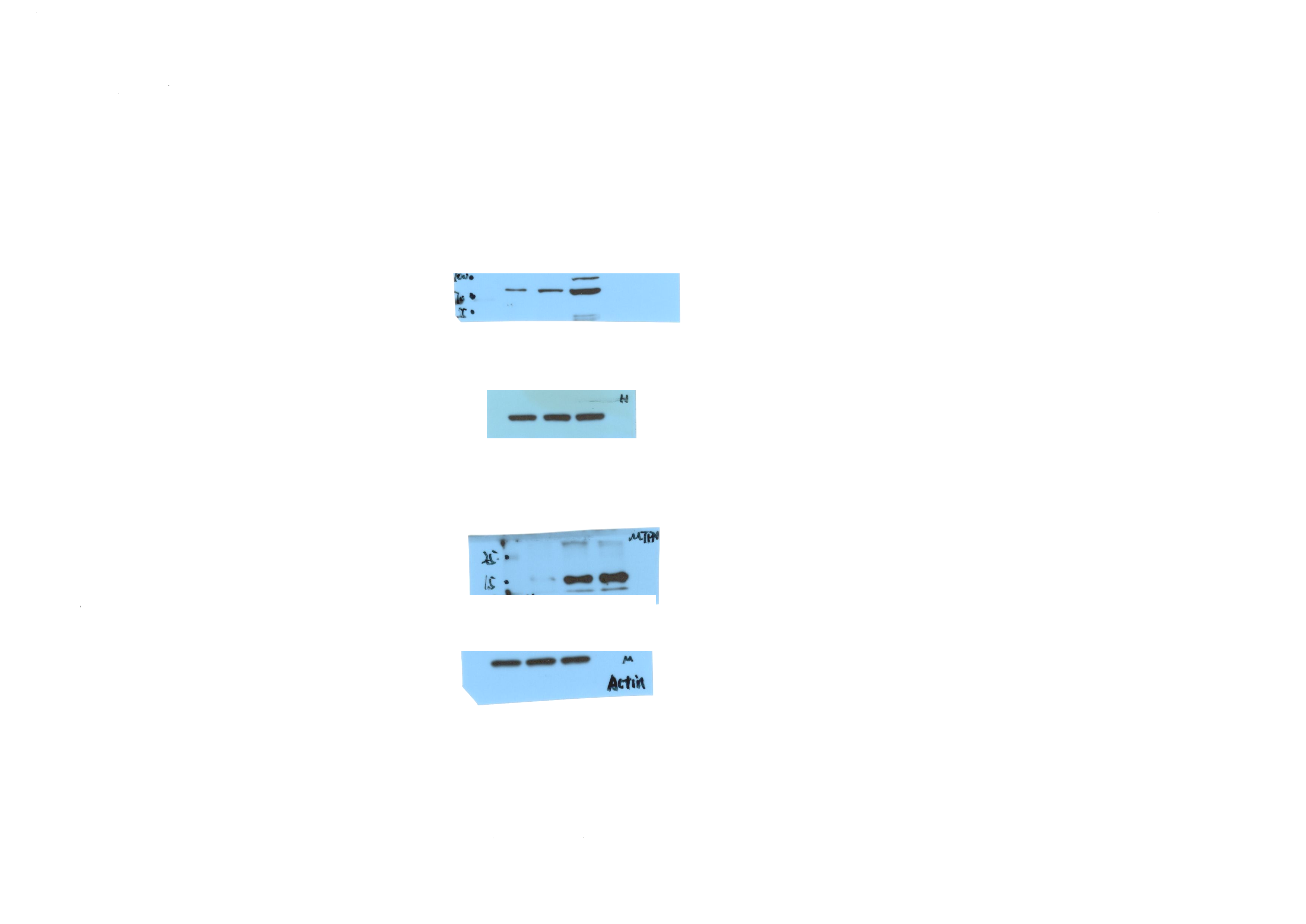

Supplement: Supplemental Information 1 [file peerj-09-11350-s001.zip › WB_revised.png]
